# The Burgeoning Importance of Nanomotion Sensors in Microbiology and Biology

**DOI:** 10.3390/bios15070455

**Published:** 2025-07-15

**Authors:** Marco Girasole, Giovanni Longo

**Affiliations:** Istituto di Struttura della Materia, Consiglio Nazionale delle Ricerche ISM-CNR, Via del Fosso del Cavaliere 100, 00133 Rome, Italy; marco.girasole@ism.cnr.it

**Keywords:** nanomotion sensor, nanomechanical sensors, bacterial resistance, antibiotic susceptibility, biological specimens: atomic force microscopy

## Abstract

Nanomotion sensors have emerged as a pivotal technology in microbiology and biology, leveraging advances in nanotechnology, microelectronics, and optics to provide a highly sensitive, label-free detection of biological activity and interactions. These sensors were first limited to nanomechanical oscillators like atomic force microscopy cantilevers, but now they are expanding into new, more intriguing setups. The idea is to convert the inherent nanoscale movements of living organisms—a direct manifestation of their metabolic activity—into measurable signals. This review highlights the evolution and diverse applications of nanomotion sensing. Key methodologies include Atomic Force Microscopy-based sensors, optical nanomotion detection, graphene drum sensors, and optical fiber-based sensors, each offering unique advantages in sensitivity, cost, and applicability. The analysis of complex nanomotion data is increasingly supported by advanced modeling and the integration of artificial intelligence and machine learning, enhancing pattern recognition and automation. The versatility and real-time, label-free nature of nanomotion sensing position it as a transformative tool that could revolutionize diagnostics, therapeutics, and fundamental biological research.

## 1. Introduction

The last decades have witnessed extraordinary advances in nanotechnology, microelectronics, and optics that have demonstrated unprecedented sensitivity for label-free identification of the activity of biological systems as well as for the detection of biological interactions. Among them, nanomechanical oscillators stand out as small, precise experimental platforms that can perform high-sensitivity analyses of nanosized biological specimens [1,2]. These sensors are designed to convert biological interactions occurring at their surfaces into measurable mechanical responses, thereby providing a highly sensitive and versatile tool for real-time biosensing without the need for labeling [3]. The evolution of nanomechanical sensors can be traced back to the early 2000s, with the first microcantilever biosensor being reported for DNA hybridization and antigen-antibody binding [4]. Since then, various types of suspended mechanical structures have been employed to quantify a wide range of analytes, including DNA, proteins, viruses, and cells.

Atomic Force Microscopes (AFMs) are commonly employed for these studies. These devices have at their core a very sensitive sensor, a cantilever, which is essentially a tiny, elastic lever with a sharp tip at the end. When the tip interacts with a surface (like bacteria, firmly attached to a substrate), it bends or deflects based on the forces involved [5]. The amount of cantilever deflection is directly proportional to the force applied by the sample. For a given applied force, a stiffer sample (like a bacterium with a strong cell wall) will cause less deflection for the same applied force compared to a softer sample [6].

Here, we will first discuss nanomechanical sensors, which are the base for the AFM-based nanomotion sensors. Next, we will introduce the nanomotion sensor, its fundamental principles, and delve into key applications in microbiology and medicine, describe novel technological modalities, and finally, consider future directions and challenges.

## 2. Nanomechanical Sensors

Since AFM cantilevers are extremely sensitive to vertical displacement, with a sensitivity in the order of 1 Å, and can be chemically functionalized to stimulate a specific molecular attachment, they are commonly used as sensors in many biological studies [7]. Particularly interesting are the ones that take advantage of their interaction sensitivity, such as those involving the attachment of single cells on their surface to perform Single Cell Force Spectroscopy [8]. Other studies exploit the displacement sensitivity of these sensors, which can be used to reveal nanoscale oscillations in living cell clusters, or even in single cells [9,10].

Even independently from an AFM, one or an array of cantilevers, coupled with a displacement detector, can result in powerful and versatile sensors [11,12] capable of characterizing biological systems with high detail and time resolution [13,14]. Overall, these nanomechanical oscillators are increasingly employed to study biological systems [15,16,17,18,19], and in particular bacteria. In a remarkable work, Kosaka and coworkers presented a sandwich assay that combines mechanical and optoplasmonic transduction to obtain a sensor capable of detecting the presence of very low concentrations of specific biomarkers in blood samples with high selectivity. The use of two different transduction mechanisms in a single platform can determine the presence of a protein with extremely high statistical significance, and this technique can translate to the development of technologies capable, through the combination with routine blood tests, of early cancer detection [20,21].

The application of these devices in the field of nanomedicine [22,23,24] has opened new avenues in the development of innovative tools in medicine. For instance, Huber and coworkers have presented an assay based on microcantilever arrays that can detect nanomechanically the mutations linked to melanoma cancer without the need for amplification in total RNA samples isolated from cells, improving the speed and sensitivity of the diagnosis [25]. Particularly important is the use of nanomechanical sensors in the investigation of bacteria and of their response to antibiotics, which is a paramount medical issue. On one side, the properties of the sensor have been optimized to study specifically these microorganisms, while on the other side, the signal collection system has been optimized to achieve a better transduction of the biologically relevant information [26,27,28]. For example, it has been shown that bacteria attached to a cantilever sensor can modify its vibration modes and that these changes depend greatly on their position on the sensor [29]. Finally, nanomechanical sensors have a large range of additional applications, and are used as artificial noses [30], for the detection of specific molecules in gaseous and fluid environments [31,32,33], or as very sensitive temperature sensors [34,35].

The size and sensitivity of nanomechanical sensors have attracted a great deal of attention with the aim of determining the viability of bacteria to perform rapid antibiotic susceptibility tests (ASTs). These studies involved the grafting of nanofluidic channels inside the sensor [36] or enhancing the sensitivity of the cantilever itself (for example, using carbon nanotubes) to detect the presence of living specimens [37,38].

The study of movement at the micro- and nanoscale of biological systems is an extremely important topic in biology, with applications ranging from oncology to microbiology. Several techniques are used to measure the movements of living specimens *in vivo* or *in vitro*, such as high-resolution microscopy, confocal microscopy, magnetic resonance interference contrast microscopy, or holographic microscopy [39,40,41,42,43]. Unfortunately, most of them require invasive procedures such as fluorescent tags [44,45,46]. On the other hand, nanomechanical sensors have been successfully used as sensors to study nanomotion in a wide range of biological entities, providing insights into the life-death transitions, activity, or metabolic states of the specimens [47].

While these nanomechanical sensors have a setup that mimics (and in some cases takes direct advantage of) the AFM setup and hardware, the outcome of these techniques is very different. AFM is used to reconstruct the morphology of a surface or to measure the mechanical properties of a sample that is attached to a surface. On the other hand, nanomechanical sensors, which are often a tip-less version of the same AFM cantilevers, study the properties of a biological material that is directly attached to the sensor itself. The outcome in one case is a quantitative map of the surface features and properties, while in the other case is a time-resolved deflection pattern that is related to the properties of the specimens attached to it.

## 3. AFM-Based Nanomotion Sensor

### 3.1. The Principle of the AFM-Based Banomotion Sensor

Most of the available nanomechanical sensor setups are limited by two major factors: they require measurements in air, and they need the bacteria to replicate directly on the sensors, measuring the resulting mass change. This is reflected in the achievable information and in the time required to perform the measurements.

To overcome these limitations, Longo and coworkers in 2011 proposed a new sensor based on AFM cantilevers, which allows measuring the nanoscale movements and vibrations of living biological systems directly in a liquid environment [48]. The principle behind this method is that living organisms exhibit continuous nanoscale vibrations as a manifestation of their metabolic activity.

**Figure 1:**
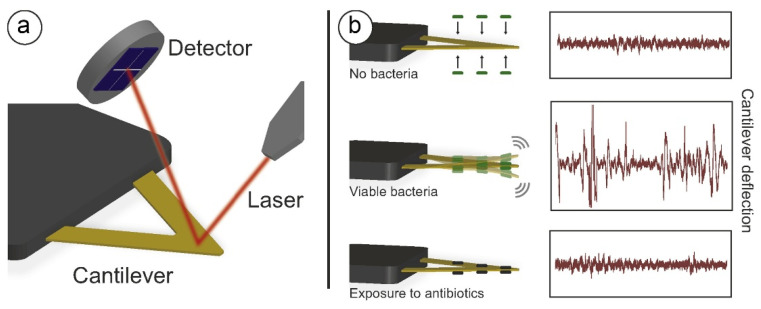
Outline of experimental setup and description of nanomotion experiments. (**a**) Schematics of the nanomotion detector setup with a cantilever sensor. (**b**) Representation of a typical nanomotion susceptibility test. When bacteria are not attached to the sensor, fluctuations are driven only by thermal motion and are low. After attachment of live bacteria, fluctuations are linked to their metabolic activity and are high. Finally, after exposure to a bactericidal drug, bacteria are nonviable, and fluctuations return to low levels. Obtained with permission from [49].

By attaching live specimens, such as cells or bacteria, to the surface of the sensor, their inherent nanoscale movements induce oscillations in the sensor, which are then precisely measured using a laser-based transduction system to transduce them into a measurable signal (Figure 1).

**Figure 2:**
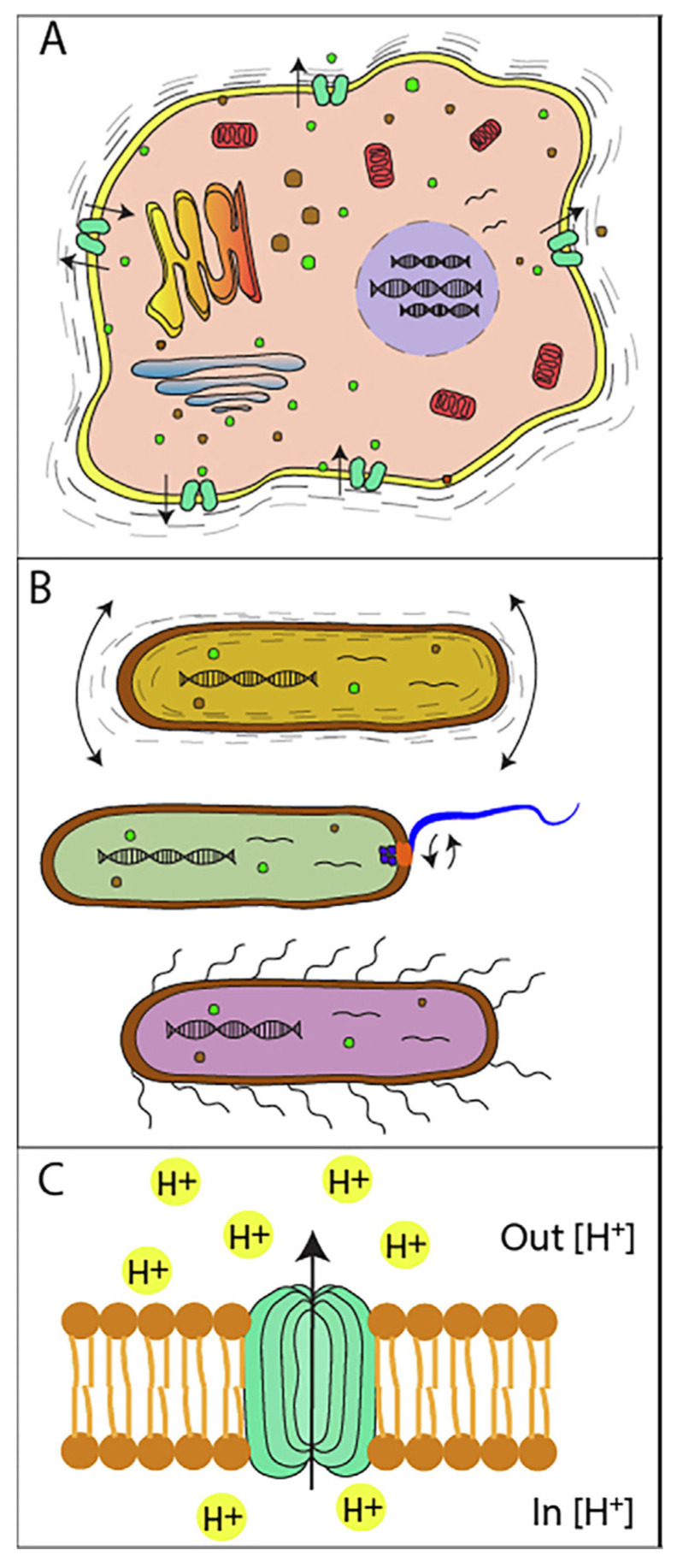
Mechanisms which can cause cell nanomotion (**A**) Molecular and cellular mechanisms in the cytoplasm could be involved in the nanomotion of a cell. Among them, mitochondria and other metabolic processes could generate motion of the overall cell. Cell membrane motion itself (as shown by the arrows) could be part of the numerous mechanisms causing cellular nanomotion. (**B**) Extracellular organelles—such as pili or flagella—are motile components used for the locomotion of cells or bacteria (the mesoscopic vibrations are depicted as arrows). This is inherent to its function, causing nanomotion of cells or bacteria. (**C**) ion channels (green) change conformation to let in or out specific ions (depicted as H+ in yellow and the arrow showing ions exiting the channel). This structure rearrangement affects the lipid bilayer of cells and could be part of the nanomotion of cells. Obtained from [54] under Creative Commons Attribution License (CC BY).

The setup of the AFM and that of the original nanomotion sensor are very similar, since they can use the same movement sensors and optical readout. Indeed, the first setups of the nanomotion sensor were carried out using commercial AFMs, but nowadays, independent and optimized systems, independent from the AFM hardware, are available. This also suggests that, as a typical AFM can detect fluctuations of the cantilever with sub-nanometer resolution, a nanomotion sensor can provide highly sensitive measurements of the biological specimen’s activity with a sensitivity that is in the same range. These oscillations are produced by the transduction of the small vibrations which are typical of any living system and reflect directly the activity of the specimens attached to the sensor. These nanoscale movements and fluctuations can be generated by specific motors (such as flagella) [50,51] or by smaller reorganizations of the cell wall, the opening of ionic channels, or even mass displacements in the cytoplasm [52,53,54] (Figure 2).

### 3.2. Atomic Force Microscopy-Based Nanomotion Antibiotic Susceptibility Test

**Figure 3:**
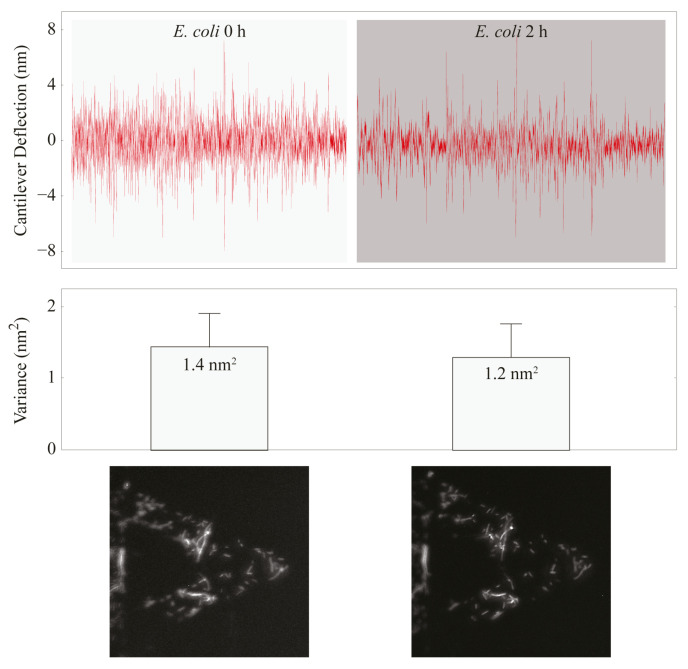
Fluorescence and bacterial movements of *Escherichia coli* before and after exposure to ampicillin. (**Top** panel): fluctuations of the cantilever. The effect of the drug is a rapid decrease in the fluctuations, indicating the death of the germs. (**Bottom** panel): fluorescence images of the cantilever. Minutes after the exposure to the drug, the fluorescence dimmed. Obtained with permission from [55].

The common feature for these movements is their association with the status and activity of the specimens under investigation. Indeed, they cease immediately upon the organism’s death, making nanomotion detection a powerful tool for assessing cellular viability [55] (Figure 3).

Since the development of the first nanomotion sensor, nanomotion has been used to study a wide range of biological entities, including bacteria, mitochondria, yeasts, and mammalian cells, tackling biological and clinical issues, and the field now encompasses several distinct techniques, each relying on different principles to detect and measure nanoscale movements. These methods offer varying levels of sensitivity, resolution, and applicability depending on the specific biological problem being investigated [53].

One of the primary applications of the AFM-based nanomotion sensor has been in rapid antibiotic susceptibility testing (AST), where the change in cantilever oscillations upon exposure to antibiotics indicates the effectiveness of the drug.

Several studies have focused on the real-time view of the bacterial response using cantilever-based nanomotion sensing. Typically, the variance of the fluctuations is used to determine the variation of the bacterial activity under different environmental conditions [50,56,57]. The amplitude of nanomotion oscillations has also been employed to study some particular behavior of the specimens, such as the formation of biofilm [58] or the virulence of the bacteria [59].

The significance of nanomotion sensing lies in its ability to provide label-free and real-time information about biological systems, without the need for prior knowledge of the specimens under investigation and for bacterial replication. This can have a vast impact in the field of microbiology [60]. For example, in a masked study by Stupar and coworkers, several bacterial species were collected from positive blood cultures and tested for susceptibility to different kinds of antibiotics.

**Figure 4:**
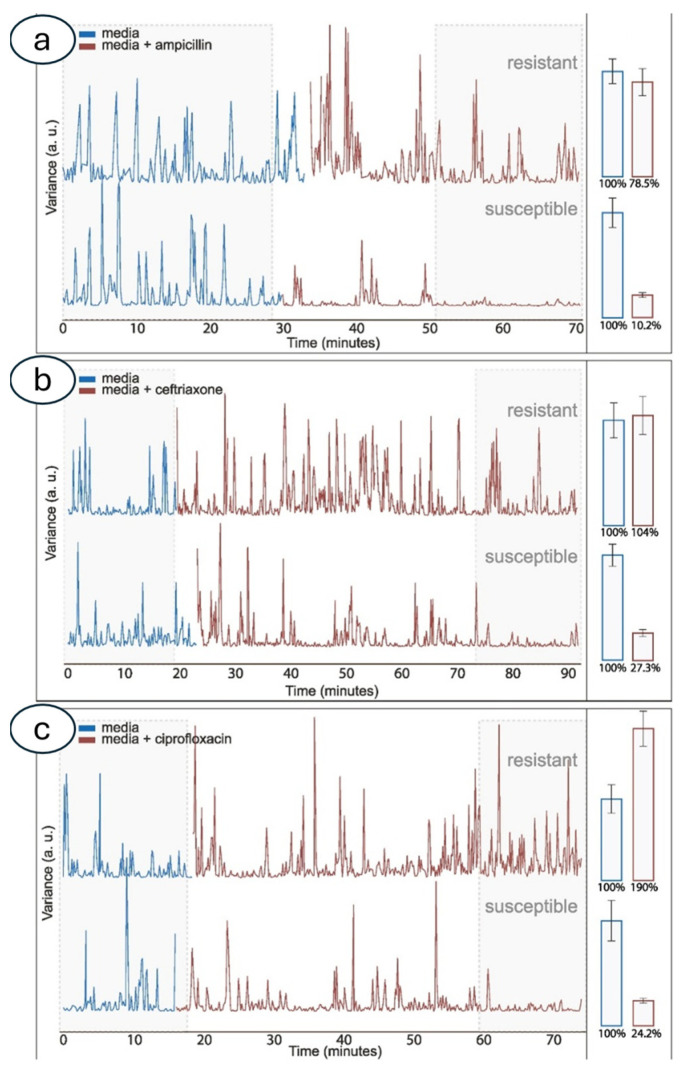
Variance evolution throughout experiments: effect of ampicillin (**a**), ceftriaxone (**b**), and ciprofloxacin (**c**) on *E. coli*. Typical examples of a resistant *E. coli* strain (top) and a susceptible strain (bottom) are presented. Blue color shows variance of the sensor’s movement while only nourishing media is present in the system. The presence of the antibiotic and the corresponding variance trend are shown in dark red. The area shaded in grey is used for averaging and presenting as change (%) on the second panels. Obtained with permission from [49].

In a few hours, the analysis of the bacterial strains yielded a success rate of 94%, which is suggestive of a possible translation of the nanomotion sensor as a new tool for microbiologists in the clinical setting [49] (Figure 4).

In fact, the advantages of this system in terms of timing and sensitivity, and especially since the susceptibility analyses are not linked to the bacterial replication time, indicate that it is of particular effect in studying slowly growing organisms. These species, such as *Mycobacterium tuberculosis* (Mtb) and nontuberculous mycobacteria (NTM), are the causative agents of extremely dangerous infections, and their slow replication time (from hours to days) makes them extremely complex to study. The first use of nanomotion sensors for the study of slowly growing bacteria was proposed by Mustazzolu and coworkers and has shown a vast reduction in time to determine the susceptibility of BCG and *Mycobacterium abscessus* compared to the commonly used phenotypic techniques (hours compared to weeks or months) [61]. This result suggests the nanomotion sensor can have a vast impact on microbiology studies of these dangerous bacteria [62].

### 3.3. Nanomotion Sensor to Study Other Biological Specimens: Mammalian Cells and Sub-Cellular Organelles

The sensitivity of the different nanomotion sensor setups has opened the way to their use to study smaller specimens. For instance, one of the first works on the cantilever-based nanomotion sensor focused on the study of Topoisomerase, determining the different steps of the protein’s rearrangement in the presence of ATP [63].

Other works have focused on sub-cellular organelles such as mitochondria. The fluctuations of these structures were measured both using the cantilever-based nanomotion [64] and ONMD [65]. This is a clear indication of the versatility of the technique and of the role that these techniques can play in biological and medical studies.

**Figure 5:**
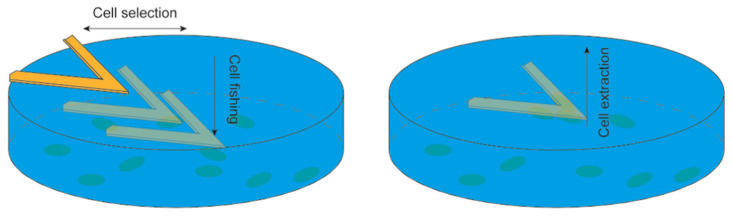
Depiction of the immobilization protocol of mammalian cells on the nanosensor. The sensor is monitored by optical microscopy to approach a cell. The chemically activated surface of the sensor is pressed over the cell for 1 min to ensure attachment and then retracted to extract the cell. This procedure is repeated to obtain 3–5 cells over a single sensor to perform the nanomotion experiment. Obtained from [70] under Creative Commons Attribution License (CC BY).

The development of nanomotion sensor setups to study bacteria and small specimens has proceeded almost concurrently with the use of this technology to study larger specimens such as mammalian cells. The study of the nanoscale movements of mammalian cells requires a more complex setup, involving controlled movements of the sensor and direct fishing of the cells in a procedure that is similar to that used in Single Cell Force Spectroscopy experiments [66]. Typically, sensors are functionalized with bio-compatible molecules, which will allow good cellular adhesion (i.e., fibronectin, APTES, poly-l-lysine, and glutaraldehyde). These are then guided and pressed on the cells to induce their adhesion to the surface of the sensor. After the cell loading, the sensor is retracted, and the cell-induced fluctuations are monitored [53] (Figure 5).

**Figure 6:**
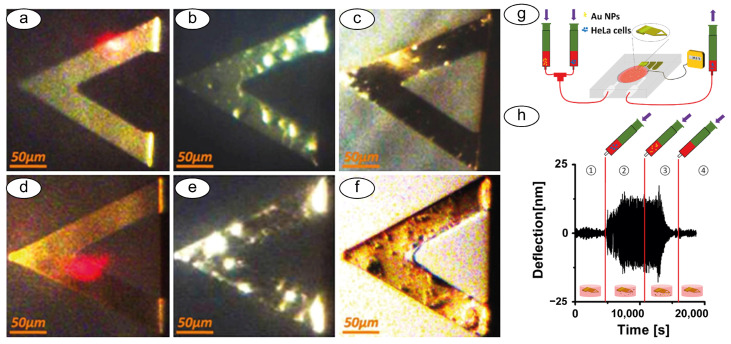
Optical images of a triangular cantilever. (**a**) Oscillating in air before measurement; (**b**) Oscillating in solution with cells adsorbed on its surface; (**c**) After washing the cantilever with ethanol and rinsing it with PBS; (**d**) In water; (**e**) After the injection of Au nanoparticles; (**f**) oscillating with absorbed cells and Au nanoparticles in air after measurement without washing; (**g**,**h**) Sketch of the analytical system and the measurement principle (1-4 the steps of cell immobilization and washing). Obtained from [67] under Creative Commons Attribution License (CC BY).

Using such a setup, researchers studied HeLa and MCF7 cells exposed to cytotoxic agents [67] (Figure 6) or neuroblastoma cell lines (SH-SY5Y) to determine the interaction of these cells with different forms of alpha-synuclein, evidencing the role of molecule aggregation in neurodegenerative diseases [68]. Lupoli and coworkers showed, through real-time nanomotion studies, the dynamics of CRISPR-based engineered cell lines (HEK-cFXN) exposed to controlled oxidative stresses, in a model of Friederich’s Ataxia, a genetic neurodegenerative disease. In this research, the nanomotion sensor was able to determine the dynamics of the cellular response to oxidative stress, shedding some light on the role of the frataxin gene [69,70]. Other studies involved the use of nanomotion sensors to evaluate the nanoscale vibrations of red blood cells during their aging and their response to energy deprivation [71].

More recently, Girasole and colleagues have used a nanomotion sensor setup to study, through optical and nanomotion investigation, the interaction between clusters of neuroblastoma cells. The dynamics of the cell clusters evidence some remarkable behavior, which was used to suggest a role for acoustic wave fields in the interaction between neuron-like cells [72].

Nanomotion sensing is also being explored for its applications in cancer research, particularly in monitoring the sensitivity of cancer cells to chemotherapeutic drugs. Similar to AST, this involves observing the changes in nanomotion patterns of cancer cells upon exposure to various anticancer agents [73]. Sensitive cancer cells typically exhibit a decrease or cessation of nanomotion as they undergo cell death or metabolic disruption, while resistant cells may continue to show significant nanoscale movements [74]. Stupar and coworkers have demonstrated how nanomotion analysis could be used to distinguish between sensitive and resistant cancer cell lines, presenting this technique as a possible game-changer in the field of personalized cancer therapy.

**Figure 7:**
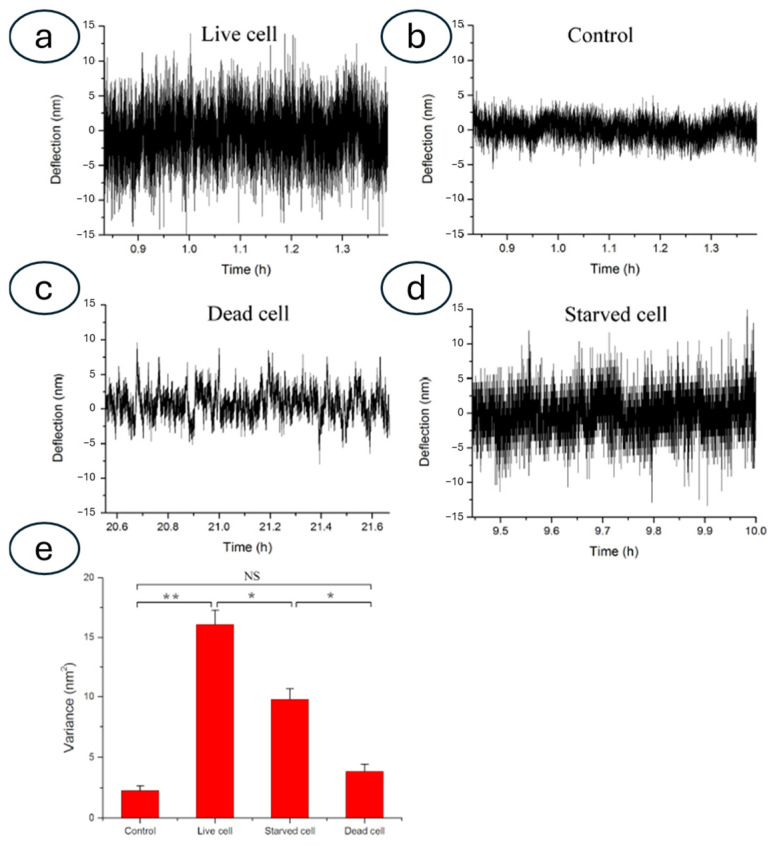
Investigation of cancer cell viability through nanomechanical fluctuations. (**a**) Nanomechanical deflection of the microcantilever with live cancer cells (MCF-7) adhered to it. (**b**) Corresponding results for the naked microcantilever without cells. (**c**) Corresponding results for the microcantilever with dead cells adhered to it. (**d**) Corresponding result for the experiment involving starved cells. (**e**) Corresponding variance values. The stars indicate the significance of the differences, while NS indicates a non-significative difference. Obtained with permission from [76].

By rapidly assessing the effectiveness of different drugs on patient-derived tumor cells, clinicians could potentially tailor treatment regimens to individual patients, maximizing therapeutic efficacy and minimizing adverse side effects [75]. Overall, nanomotion sensing offers a label-free and real-time approach to evaluating drug responses in cancer cells, providing valuable information that can guide treatment decisions and accelerate the development of new anticancer therapies [76] (Figure 7).

The prospect of using nanomotion sensing to rapidly evaluate the effectiveness of various cancer treatments on a patient-specific basis has the capacity to transfer to the field of personalized oncology. Current methods for assessing drug sensitivity in cancer cells can be time-consuming and may not fully capture the dynamic interactions between drugs and cells. Nanomotion sensing offers a faster and potentially more accurate way to determine how a patient’s cancer cells respond to different therapeutic agents, paving the way for more individualized and effective treatment strategies.

## 4. Novel Setups

### 4.1. Optical Nanomotion

Optical Nanomotion Detection (ONMD) presents a simpler and cost-effective alternative to AFM for studying micron and sub-micron scale biological movements. This technique utilizes a traditional optical microscope equipped with a standard video camera to record the motion of biological samples, typically cells, deposited on a glass surface [73]. The optical images are collected over a period of time with a lateral resolution that allows a clear identification of the vibrations and movements of the specimens. These movements are then analyzed using a custom image software, which is used to identify and track the two-dimensional displacements of these cells with sub-pixel resolution, enabling the detection of nanoscale oscillations [77].

**Figure 8:**
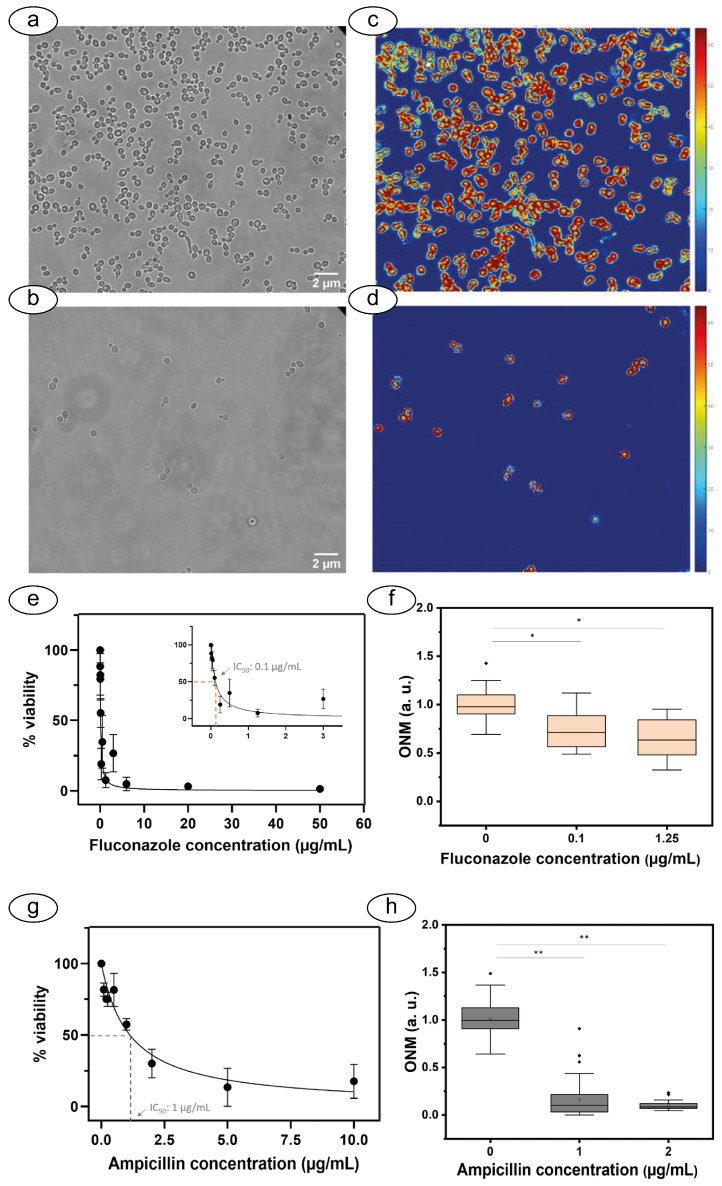
Example of optical nanomotion: Optical (**a**,**b**) and images highlighting the difference between the first and last image (**c**,**d**) of *Candida albicans*. In buffer (**a**,**c**) and exposed to fluconazole (**b**,**d**). Viability curves using conventional OD600 assays (**e**,**g**) and the ONMD boxplots (**f**,**h**) for *C. albicans* (upper panels) and *E. coli* (bottom panels) exposed to fluconazole and ampicillin, respectively. The stars indicate the significance of the differences. Obtained from [78] under Creative Commons Attribution License (CC BY).

ONMD offers several advantages over AFM-based methods. It does not require the attachment of cells to a cantilever, simplifying sample preparation and making it suitable for studying both single cells and entire populations. The use of standard optical microscopy equipment makes ONMD significantly cheaper and easier to implement, potentially broadening its accessibility. This technique has been successfully applied by Villalba and coworkers to rapid AST for bacteria and yeasts, demonstrating its ability to effectively determine antimicrobial susceptibility within a short timeframe, also allowing the concurrent measurement of the movements and vibrations of many specimens [78] (Figure 8). The simplicity and affordability of ONMD hold the potential to make this a viable option for routine diagnostics and research in diverse settings, including resource-limited environments, at the cost of a reduced sensitivity to smaller movements, which are below the resolution limit of optical imaging. This increased accessibility could lead to a wider adoption of this technology, facilitating its use in a broader range of biological and medical applications, ultimately benefiting both research and healthcare in various parts of the world.

**Figure 9:**
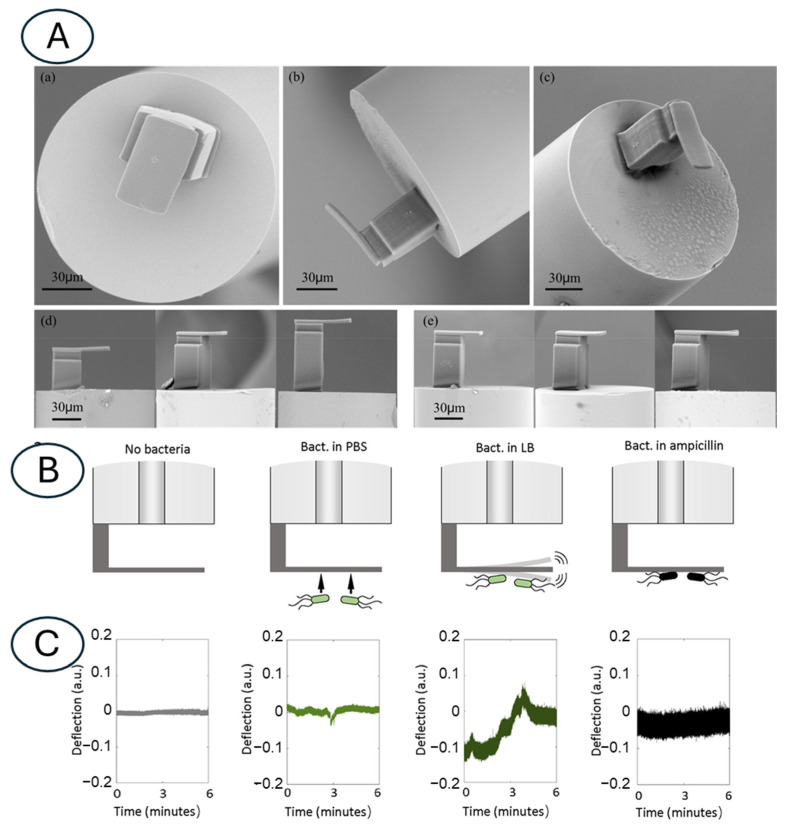
(**A**) Scanning electron microscopy image viewed from different angles of the polymer cantilever on the fiber tip fabricated using a two-photon polymerization technique. (**a**–**c**) The polymer μ-cantilever viewed from different angles, showing the details of the μ-cantilever on the 125 μm diameter optical fiber. The edge of the base is 10 μm away from the core to improve the reflectivity without affecting the sensitivity. (**d**) Polymer μ-cantilevers with different base heights (40, 60, and 80 μm). (**e**) Polymer μ-cantilevers with different lengths (20, 30, and 40 μm). (**B**) Schematic representation of the nanomotion detection assay of *E. coli* susceptible to ampicillin by optical fiber nanomotion sensor. (**C**) Representative deflection signals of the cantilever in culture medium (gray), during the attachment procedure (light green), and of the cantilever with attached *E. coli* before (green) and after drug exposure (black). Obtained from [80,81] under Creative Commons Attribution License (CC BY).

Another promising avenue in nanomotion sensing involves the use of optical fiber-based sensors. These sensors typically consist of a microfabricated cantilever that is aligned [79] or attached to the distal end of an optical fiber [80]. The oscillation of this cantilever, induced by the nanomotion of attached microorganisms, is monitored using in-fiber interferometry, allowing the monitoring of the cantilever’s displacement with very high sensitivity. Researchers have demonstrated the excellent performance of these sensors in susceptibility tests with bacteria like *E. coli* and fungi like *C. albicans*, achieving fast response times in the range of minutes [81] (Figure 9).

Optical fiber-based nanomotion sensors offer several advantages, including their compact size, potential for parallelization, and high sensitivity. The simplicity of use and the ability to create sensor arrays would make them promising candidates for future high-throughput diagnostic applications.

### 4.2. Optical Cavities and Graphene Membranes

**Figure 10:**
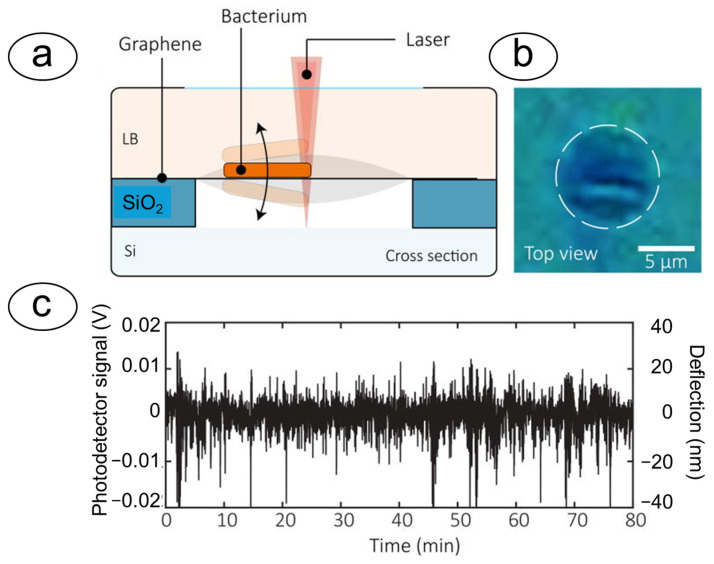
Schematization of graphene drums nanomotion: (**a**) Schematics of graphene drums. (**b**) Optical image of a single bacterial cell attached to a graphene drum. The drum is outlined by a dashed white circle. (**c**) Nanomotion signal obtained by measuring a single cell under constant conditions for more than an hour. Large oscillations occur with an amplitude of up to 40 nm. Obtained from [82] under Creative Commons Attribution License (CC BY).

Graphene, a two-dimensional material with exceptional mechanical and electrical properties, has emerged as a promising material for highly sensitive nanomotion sensors. These sensors typically consist of an ultra-thin suspended graphene membrane, often in the form of a drum, which vibrates in response to the minute forces exerted by attached microorganisms. The displacement of the graphene drum is then measured with high precision using laser interferometry [82]. The key advantage of graphene-based sensors lies in their extremely low stiffness, which allows them to transduce even picoNewton-scale forces, making them highly sensitive to the nanomotion of single cells. This capability is particularly valuable for probing life at the nanoscale and for applications such as rapid AST, where the response of individual bacteria to antibiotics can be monitored in real-time. Graphene drums have shown the ability to detect nanomotion signals from clinical isolates of various bacterial species, highlighting their possible use in clinical diagnostic settings [51] (Figure 10).

In a similar fashion, some works are now emerging, which describe the use of optical traps to study bacterial motion at the single cell level and with sensitivity and selectivity to define the bacterial Gram-type [83] or even investigate single viruses [84]. These studies show some innovative setups that combine microfluidics and optical cavities to obtain a reusable tool that can be easily multiplexed on single chips to obtain parallel, fast, and sensitive analysis. These devices have been employed to perform several studies on bacteria exposed to antibiotics or to phages, which represent a promising avenue to follow to solve the issue of bacterial resistance [85].

The exceptional sensitivity of these setups for single-cell analysis opens possibilities for gaining a deeper understanding of individual cell behavior and the heterogeneity within cell populations. By studying the unique nanomotion signatures of individual cells, researchers can potentially uncover novel insights into disease mechanisms, drug responses, and other biological phenomena that might be masked when analyzing bulk populations. This level of detail could be crucial for advancing our knowledge of complex biological systems and developing more targeted therapies.

## 5. Models, Big Data, and Integration with Artificial Intelligence

**Figure 11:**
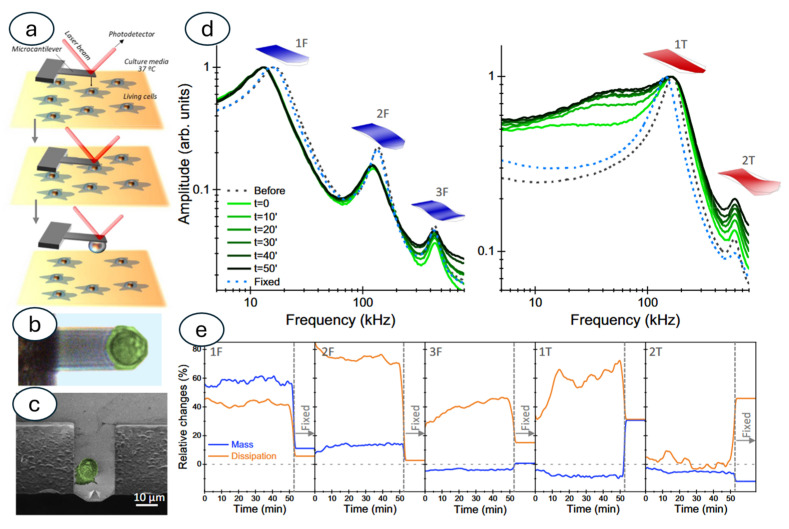
Experimental realization and data analysis for unveiling mechanical resonances of living human cells in physiological conditions. (**a**) Schematics of the cell capture by the cantilever. (**b**) Optical image of an MCF-10A breast epithelial cell adhered to the cantilever surface. (**c**) Scanning electron microscopy of an MCF-10A cell after fixation. (**d**) Frequency spectra of the flexural-like and torsional-like thermal displacement fluctuations of the cantilever before and during the cell attachment, and after cell fixation. The first flexural vibration modes are labeled as 1F, 2F, and 3F, and the first torsional vibration modes are labeled as 1T and 2T. (**e**) Fractional changes of the apparent mass added to the cantilever and energy dissipation of the system due to internal losses of the cell. Obtained from [91] under Creative Commons Attribution License (CC BY).

Beyond diagnostics, nanomotion sensing is proving to be a valuable tool in fundamental biological research. It provides a unique way to investigate the link between nanomotion and the metabolic activity of living cells. Since the nanomotion data are a very large set of time-related oscillations, the analysis of this information is extremely complex. Research has started from the basic understanding of the fluctuations of a cantilever in a liquid environment. This has been performed through models [86], finite element simulations [48,87], by forced oscillations of the sensor [88], or by controlled loading of cells on the sensor’s surface [29], to evaluate the changes in properties. Even the position of the specimens on the sensor has been evaluated from a theoretical point of view to understand its role in optimizing the transduction [89,90]. In fact, finite element modeling allows evaluating the oscillation patterns produced by living biological systems on a sensor. This is shown by Puerto-Belda and coworkers, where a model of the different modes of oscillation of a sensor has been used to determine the vibrational properties of the cells attached to the sensor [91] (Figure 11).

**Figure 12:**
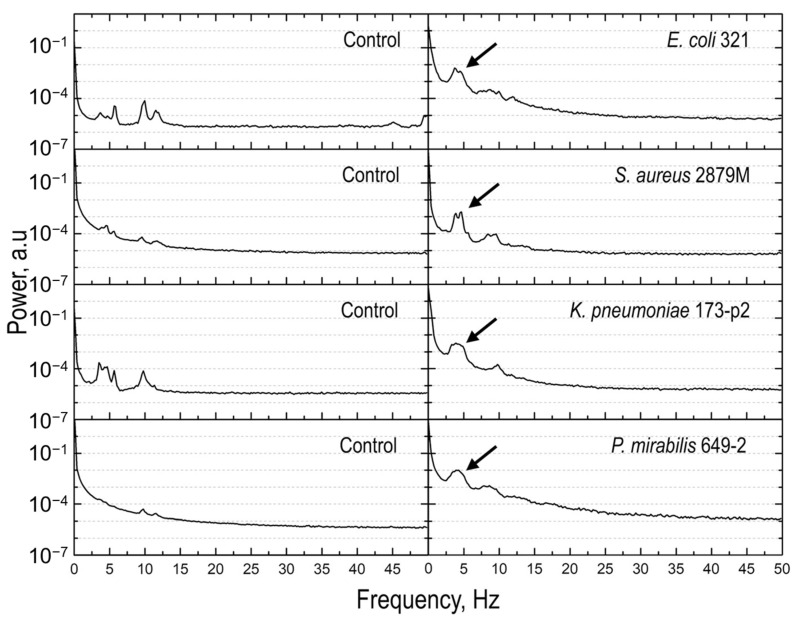
Time-dependent relationships (power vs. frequency) in control (empty cantilever) and bacteria (cantilever with different bacterial strains attached) experiments. The arrows represent the ~2 Hz features in the spectra which are representative of the bacterium under investigation. Obtained from [93] under Creative Commons Attribution License (CC BY).

**Figure 13:**
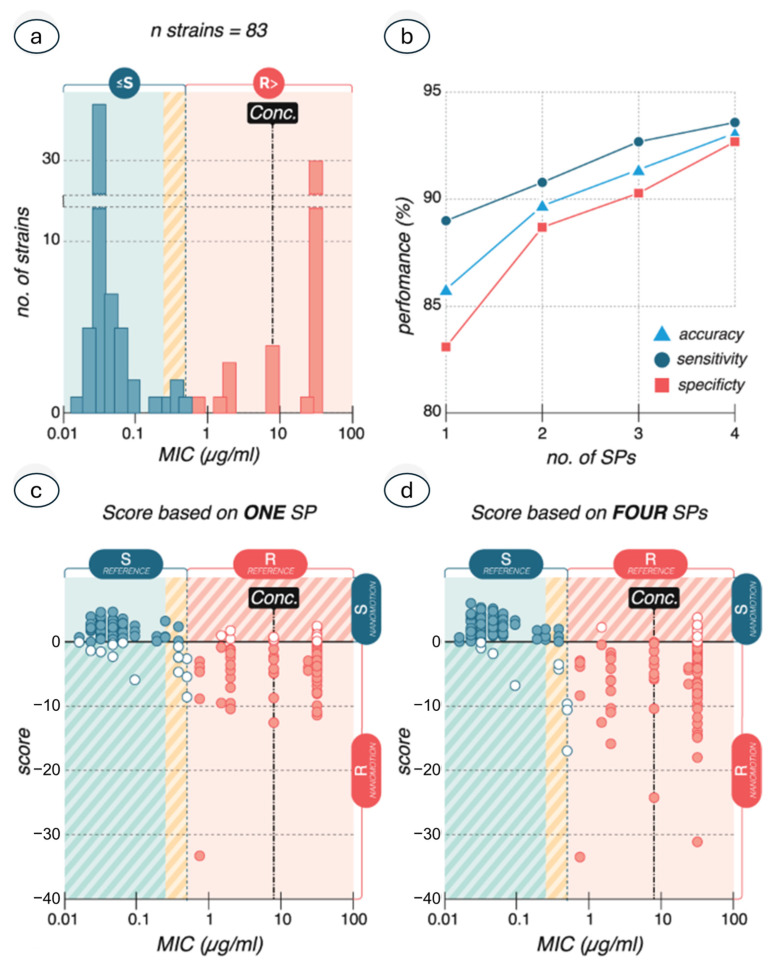
(**a**) The MIC distribution of *K. pneumoniae* isolates used to train classification models. The dotted line represents the border between the susceptible (≤S) and resistant (R>) classes, EUCAST (2022). The yellow shaded area represents the “susceptible increased exposure category” combined with the S class (green) in nanomotion AST. (**b**) Classification of model accuracy, sensitivity, and specificity when training the AI using a different number of signal parameters (SPs). (**c**) Classification according to nanomotion AST based on one SP. Each circle represents a single nanomotion measurement for which a score was calculated with the two classes defined as S > 0 > R. Closed circles show correctly classified measurements [True Positive (TP, correctly classified susceptible isolates), True Negative (TN, correctly classified resistant isolates)], and open circles show falsely classified measurements [False Positive (FP, falsely classified resistant isolates), False Negative (FN, falsely classified susceptible isolates)]. (**d**) Classification according to nanomotion AST based on four SPs. The number of falsely classified experiments decreased. Obtained from [94] under Creative Commons Attribution License (CC BY).

Some works have limited their focus on the variance of the oscillations, but others are introducing more detailed analyses, such as the determination of the frequency content of the oscillations and the modeling of the fluctuations, to help determine the behavior of the specimens with higher confidence [92]. Indeed, in several cases, the mere study of the variance of the oscillations does not allow a complete overview of the information content of the nanoscale vibrations, and other mathematical analyses must be taken into consideration [93]. This includes a frequency deconvolution of the nanomotion signal and the evaluation of the power density spectrum (Figure 12) that allows understanding how the distribution of energy delivered by the biosystems is organized. In this sense, the integration of nanomotion sensing with artificial intelligence and machine learning algorithms can become highly beneficial. Indeed, AI algorithms can be trained on large datasets of nanomotion signatures to automatically classify bacterial species or predict antibiotic resistance profiles with higher accuracy. This would allow them to recognize complex patterns in nanomotion signals, enabling a more accurate and rapid classification of biological samples, such as distinguishing between antibiotic-resistant and susceptible bacteria. In this sense, Sturm and collaborators showed that machine learning allows performing rapidly and with high accuracy ASTs on different bacteria, such as *Klebsiella pneumoniae*, highlighting the role of training in the identification efficiency (Figure 13) [94] Similar semi-automated techniques are extremely useful to perform fast AST in a non-supervised manner and automating the interpretation of large nanomotion datasets, such as for slowly growing bacteria [95], thus improving the efficiency of diagnostics and drug screening.

## 6. Conclusions

In conclusion, nanomotion sensing has emerged as a transformative technology with significant potential to advance our understanding of biological and biomedical problems. Anticipated short-term advancements include continued improvements in sensor sensitivity, resolution, speed, and miniaturization, leading to increased adoption in rapid diagnostics, particularly for infectious diseases.

A remarkable point in favor of the nanomotion sensor is its versatility and the ability to be combined with other investigation tools, such as high-resolution microscopy or fluorescence microscopy, to correlate nanoscale movements with cellular structure and dynamics. This interdisciplinary approach enables the real-time visualization of cellular processes associated with nanomotion, providing a more comprehensive understanding of biological phenomena at the nanoscale. The growing integration with microfluidics and artificial intelligence will enable automated and high-throughput applications, further expanding the utility of this technology. This integration can lead to the development of semi-automated, high-throughput platforms for drug screening and diagnostics, as well as enabling single-cell analysis and studies in controlled microenvironments.

In the long term, nanomotion sensing holds immense promise for application in personalized medicine, allowing for tailored drug therapies and more informed treatment decisions. It will also continue to contribute significantly to fundamental biological research, providing new tools to understand complex cellular processes and the very nature of life at the nanoscale. The highlight of nanomotion sensing lies in its ability to revolutionize infectious disease diagnostics and management through rapid AST, enable personalized cancer therapy, accelerate drug discovery, and provide new avenues for exploring the fundamental principles of life. As technology continues to mature and interdisciplinary collaborations flourish, nanomotion sensing is poised to make significant contributions to both our understanding of the living world and our ability to combat disease.

## Data Availability

No original data were produced for this work.

## Abbreviations

The following abbreviations are used in this manuscript:

AFM	Atomic Force Microscope
AST	Antibiotic Susceptibility Test
ONMD	Optical Nano Motion Detection
